# Synthesized soliton crystals

**DOI:** 10.1038/s41467-021-23172-2

**Published:** 2021-05-26

**Authors:** Zhizhou Lu, Hao-Jing Chen, Weiqiang Wang, Lu Yao, Yang Wang, Yan Yu, B. E. Little, S. T. Chu, Qihuang Gong, Wei Zhao, Xu Yi, Yun-Feng Xiao, Wenfu Zhang

**Affiliations:** 1 State Key Laboratory of Transient Optics and Photonics, Xi’an Institute of Optics and Precision Mechanics, Chinese Academy of Sciences, Xi’an, China; 2 State Key Laboratory for Mesoscopic Physics and Frontiers Science Center for Nano-optoelectronics, School of Physics, Peking University, Beijing, China; 3 University of Chinese Academy of Sciences, Beijing, China; 4 Department of Physics and Materials Science, City University of Hong Kong, Kowloon Tong, Hong Kong; 5 Collaborative Innovation Center of Quantum Matter, Beijing, China; 6 Collaborative Innovation Center of Extreme Optics, Shanxi University, Taiyuan, China; 7 Department of Electrical and Computer Engineering, University of Virginia, Charlottesville, VA USA; 8 Department of Physics, University of Virginia, Charlottesville, VA USA

**Keywords:** Nonlinear optics, Solitons

## Abstract

Dissipative Kerr soliton (DKS) featuring broadband coherent frequency comb with compact size and low power consumption, provides an unparalleled tool for nonlinear physics investigation and precise measurement applications. However, the complex nonlinear dynamics generally leads to stochastic soliton formation process and makes it highly challenging to manipulate soliton number and temporal distribution in the microcavity. Here, synthesized and reconfigurable soliton crystals (SCs) are demonstrated by constructing a periodic intra-cavity potential field, which allows deterministic SCs synthesis with soliton numbers from 1 to 32 in a monolithic integrated microcavity. The ordered temporal distribution coherently enhanced the soliton crystal comb lines power up to 3 orders of magnitude in comparison to the single-soliton state. The interaction between the traveling potential field and the soliton crystals creates periodic forces on soliton and results in forced soliton oscillation. Our work paves the way to effectively manipulate cavity solitons. The demonstrated synthesized SCs offer reconfigurable temporal and spectral profiles, which provide compelling advantages for practical applications such as photonic radar, satellite communication and radio-frequency filter.

## Abstract (web summary)

Here the authors demonstrate an on-demand generation of perfect soliton crystal using synthesized potential field. The individual solitons can also be controlled, for example oscillate around their equilibrium position, by the external field.

## Introduction

Dissipative Kerr solitons (DKSs) are self-organized wave packets in photonic-chip-based microcavities^1–7^, where the chromatic dispersion is balanced by Kerr nonlinear shift, and the cavity dissipation is offset by Kerr parametric gain. DKSs produce ultrashort pulse trains and equally spaced comb lines^8–15^, and have attracted significant interest for applications, including optical communications^16,17^, spectroscopy^18,19^, ultrafast ranging^20–23^, low-noise microwave generation^24^, frequency synthesis^25^/division^26^, and quantum key distribution (QKD)^27^, etc. Up to date, various soliton states have been theoretically and experimentally demonstrated, such as Stokes solitons^28^, Brillouin-Kerr solitons^29^, breather solitons^30–34^, laser cavity solitons^35^ as well as soliton crystals (SC)^36–39^. Particularly, perfect soliton crystals (PSC)^38,39^, where temporal solitons are equally spaced in the microresonator, are of great interest. While preserving many characteristics from the single-soliton state, *i.e*., smooth spectral envelope and highly ordered temporal soliton distribution, they offer higher comb power per line and flexible soliton repetition rate, which are critical for microcomb applications. Previously, the generation and stabilization of SC rely on avoided mode crossing (AMX) induced background potential field^36,38^ or delicate control of the pump condition^39^. However, the above schemes both have complex dynamics processes and require extreme fabrication precision to reproduce or demand material-specific properties, which cannot offer a wide-range, universal switching for SC states.

In this article, we propose and demonstrate on-demand synthesis and manipulation of a library of PSCs through a controlled potential field. The *N*-period modulated background potential field is constructed by pumping the cavity with an additional control light at the frequency which is *N*-mode spaced from the primary pump light. As the period and intensity of the potential field can be conveniently tuned by the control light, our method offers full reconfigurable capability to the PSCs generation. We demonstrate synthesized SCs with a given soliton number *N* from 1 to 32 on demand, equivalent to a reconfigurable soliton generator with a repetition rate ranging from ~49 GHz to ~1.57  THz. Furthermore, the group velocity mismatch between the SC and potential field^40,41^ are investigated both theoretically and experimentally, which induces forced oscillation in the power and intracavity position of the synthesized SCs^42^. Finally, we show that the repetition rate of the synthesized SC changes linearly with the relative velocity between the potential field and the SC. The mechanism shown in this work can be extended to other platforms to greatly enhance the flexibility of on-chip SC for practical applications.

## Results

### Synthesized SCs

**Fig. 1:**
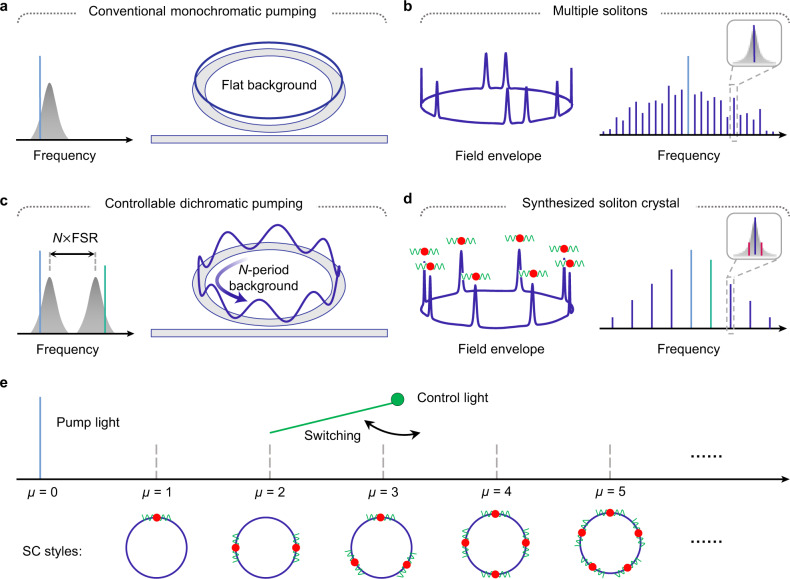
Conceptual schematic for deterministic SC synthesis and switching in the microcavity. **a** Conventional monochromatic pump scheme where solitons are initialized from a flat CW background, which generally results in a multiple-soliton state with a stochastic angular distribution of the solitons and accompanying irregular spectral envelope, such as an 8-soliton state shown in (**b**). Inset: single comb line exists in each resonance. **c** Proposed deterministic SC synthesis scheme by introducing an additional control light. The beating of the pump light (blue line) and the control light (green line) constructs a traveling periodic modulated background and draws the soliton into the equally spaced potential wells. To create *N* soliton crystal, the frequency of the control light shall be *N* free spectral range (FSR) away from the pump light. **d** Synthesized SC in the dichromatic pumped system with equal temporal spacing, which corresponds to a smooth *sech*^2^ envelope in the spectrum. The vibration of soliton will induce modulated sidebands around the main comb lines (inset). **e** By switching the control light at different mode number *μ* (*μ* = 0 for pump mode), the styles of the SC could be reconfigured on demand.

**Fig. 2:**
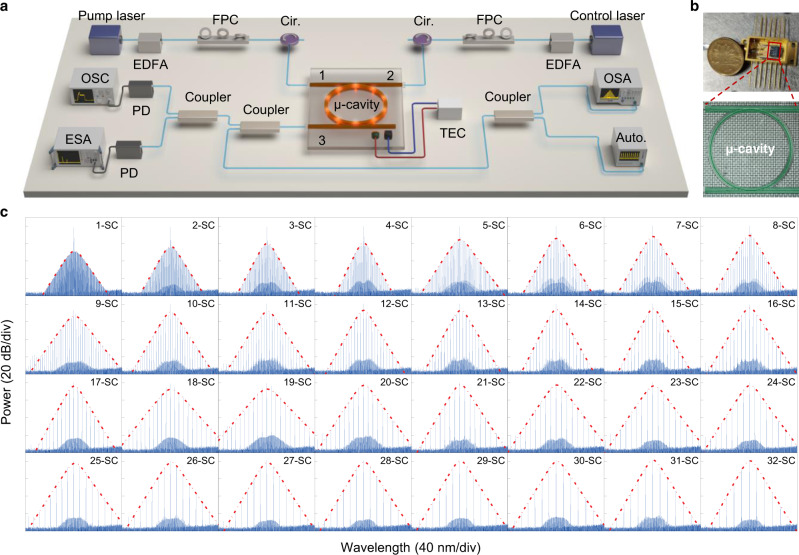
A library of 1–32 soliton crystals. **a** Illustration of the experimental set-up, the wavelength of the pump light is fixed, while the wavelength of the control light is tunable for both SC switching and intracavity thermal balancing. EDFA erbium-doped fiber amplifier, FPC fiber polarization controller, Cir. circulator, PD photodiode, TEC thermoelectric cooler, OSC oscilloscope, ESA electric spectrum analyzer, OSA optical spectrum analyzer, Auto. autocorrelator. **b** Butterfly-packaged device with a 20.5-mm-diameter Chinese coin for comparison (upper panel). Microscope image of the high-index doped silica glass microring resonators with a diameter of ~1.2 mm (lower panel). **c** Complete optical spectra for 1–32 synthesized SC with smooth *sech*^2^ envelope (red dashed line).

In a conventional monochromatic pumping scheme, the cavity soliton is initialized from a flat CW background field (Fig. 1a). The uniformly distributed gain makes the positions and quantity of the generated solitons unpredictable^43^, resulting in irregular multiple soliton pulse train and unsmooth spectral envelope (Fig. 1b). In our approach, a second CW laser is introduced as a control light to provide a periodically modulated CW background from the two-beam beating and control the SC generation process. The periodic number of potential field *N* is equal to the mode number spacing between the primary pump light and control light. Figure 2a shows the experimental setup for SC synthesis. The pump laser and the control laser are amplified and coupled to a single high-*Q* microcavity resonance from two counter-propagating directions via two fiber circulators. The role of the control laser is twofold. On the one hand, a small portion of the control light is back-scattered to the opposite direction and beats with the co-propagating clockwise pump light, constructing a periodically modulated background field. On the other hand, the main part of the control light propagating in the counter-clockwise direction is adopted to stabilize the cavity temperature when the pump light transitions into the red-detuned regime^40,44^. The add-drop type microcavity is fabricated on a CMOS-compatible high-index doped silica glass platform^45,46^. The *Q* factor and free spectral range (FSR) of the microresonator are ~2.63 million and ~48.9 GHz, respectively. To couple the light onto the chip, a standard 250 μm pitch fiber array glued to the on-chip bus waveguide is used^47,48^. The microcavity is butterfly-packaged with a thermo-electric cooler (TEC) which is controlled by an external controller^12,37^.

**Fig. 3:**
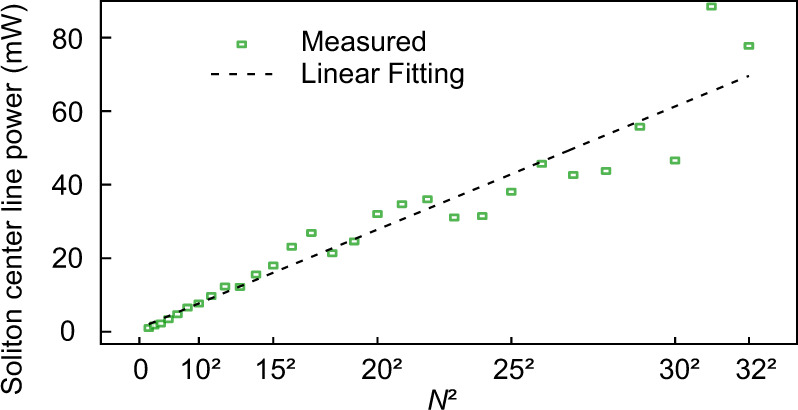
Enhancement of soliton comb line power. The soliton center line power extracted from experimental spetral envolopes versus square of soliton number *N*^2^, which shows *N*^2^ enhancement in comparison to the single-soliton state excited under similar pump conditions.

In the experiment, SC can be generated reliably by slowly decreasing the operating temperature through the TEC controller to sweep cavity resonance from blue-detuned to the red-detuned regime. The temporal and spectral profiles are measured using an autocorrelator and an optical spectrum analyzer (OSA), respectively. By switching the mode number spacing between the control and pump light, a library of 1–32 SC with repetition rates from 1× to 32 × FSR of the microcavity, are deterministically realized with high repeatability (see section 3.1 of SI for more experimental evidence). As shown in Fig. 2c, the measured spectra are all smooth with a *sech*^2^ envelope and the comb line power is enhanced by *N*^2^ (Fig. 3), thanks to the ordered distribution of the synthesized SC^38^. Moreover, the spatial overlap between the CW pump light and the soliton pulses becomes larger with the increasing soliton number, providing great potential to boost the soliton conversion efficiency. The demonstrated reconfigurable and high-power SC in a monolithic microcavity provides an ideal toolroom for tunable microwave/millimeter-wave photonics^49,50^.

**Fig. 4:**
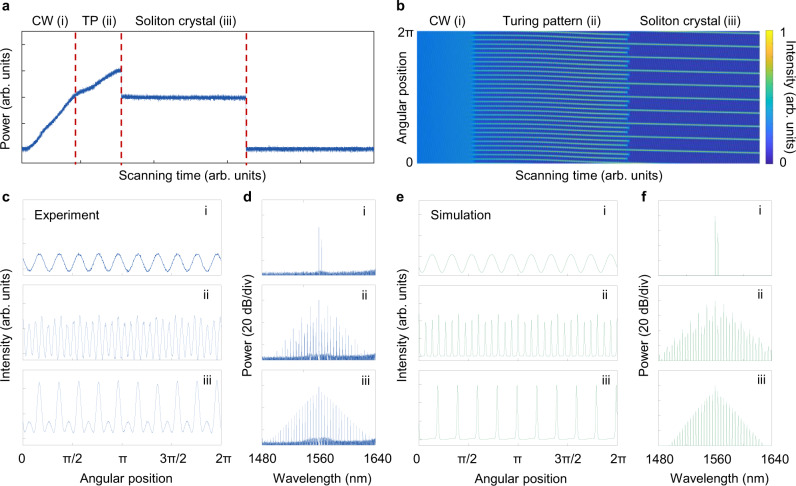
Formation dynamics of soliton crystal. **a** Measured intracavity power evolution trace. With cavity resonance scanning from longer wavelength to shorter wavelength gradually, the laser-cavity detuning varies from blue detuned to red-detuned regime. Three states are respectively marked as: state i, CW background; state ii, modulated Turing pattern (TP); state iii, soliton crystal (SC). The deterministic "step” in the trace is a characteristic feature of SC formation. **b** Simulations of the intracavity temporal waveform evolution. Turing combs directly turns to SC state as the pump enters red-detuned regime. **c**, **d** Measured temporal (**c**) and spectral (**d**) profile in different states: modulated CW background (i), modulated TP (ii), SC (iii). **e**, **f** Simulated angular (**e**) and spectral (**f**) profile in different states, showing good agreement with the experiment.

We then focus on understanding the formation dynamics of the SC by taking 10-SC as an example. The wavelength of the pump and control light are 1560.2 and 1564.1986 nm (~10 FSR away from pump), respectively. The evolution of the power is recorded while the temperature of the microcavity is gradually decreased to scan the pump-cavity resonances from longer wavelength to shorter wavelength side (Fig. 4a). Three typical stages are clearly recognized: (i) CW, (ii) modulated Turing pattern (TP) and (iii) SC. These stages are characterized by simultaneously measuring the intracavity optical field in temporal (Fig. 4c) and spectral (Fig. 4d) domain. The SC formation is recognized by the characteristic “step” power trace, ordered temporal pulse distribution, as well as the smooth *sech*^2^ spectral envelope. The soliton formation is able to bypass the chaotic regime, and is directly evolved from the modulated TP stage. The modulation field causes the multiple Turing pulses turn into *N* equally spaced solitons, i.e., *N*-SC. The evolution process of sythesized SC is well reproduced by numerical simulation of dichromatic-pump Lugiato-Lefever equation (DLLE, see SI for details). When the pump detuning is gradually increased, the three stages discussed above are sequentially distinguished, as shown in the simulated power trace in the temporal evolution trace in Fig. 4b. The simulated temporal waveforms and spectra of the three representative stages are shown in Fig. 4e, f, which agree well with the experiment.

### Forced oscillation of SCs

In the course of generating synthesized SCs, a novel type of soliton oscillation is observed when the modulated CW background field has a relative velocity to SC. This relative motion of the modulated background field creates an moving offset (phase difference) between the potential field and the SC, which provides a periodic force on the soliton^51^. Such force is derived as (See SI for details):

1 $$F={F}_{0}\cos (\eta {\phi }_{c}-{{\Delta }}\widetilde{\omega }\tau )$$

**Fig. 5:**
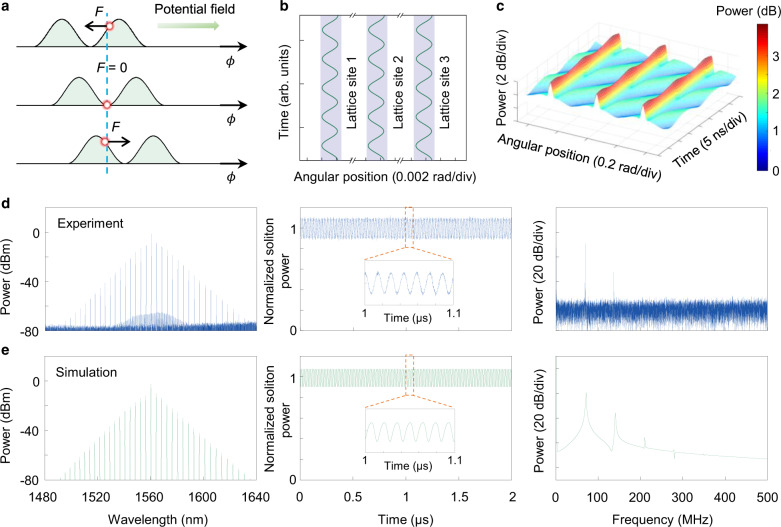
Forced oscillation of SC. **a** Illustration showing force applied on SC. The moving potential field imposes a periodic force on soliton pulses, resulting in the forced oscillatory motion of SC. **b** Simulated intracavity angular position oscillation of 3 lattice sites from a 10-SC state. Simulated intracavity field envelope is shown in (**c**). The modulated background has a relative moving speed to SC, and SC vibrates around its equilibrium position with oscillating power. **d**, **e** Experimental (**d**) and simulated (**e**) optical spectrum (left panel), normalized soliton power trace (middle panel, inset: zoom-in of 1–1.1 μs range) and electrical spectrum (right panel) for 10-SC state. Electrical spectra show that the fundamental oscillation frequency is 69 MHz.

Here *F*_0_ is the amplitude of *F*, which is proportional to the amplitude of control light. *η* = *μ*_*c*_ − *μ*_*p*_ is the mode number spacing between the pump light and control light. *ϕ*_*c*_ is the angular position of soliton center, *τ* is the normalized slow time. ${{\Delta }}\widetilde{\omega }=2({\omega }_{c}-{\omega }_{p}-\eta {D}_{1})/\kappa$ is the normalized mismatch angular frequency. As a result, the SCs oscillate around their equilibrium positions periodically (Fig. 5a). To gain more insights into the intracavity dynamics, we first define the soliton momentum as (see SI for details): *P* = 2*β*∑_*μ*_*μ*∣*ψ*_*μ*_∣^2^, here 2*βμ* and ∣*ψ*_*μ*_∣^2^ represent the normalized relative group velocity and photon number of mode *μ*, respectively. Based on the momentum analysis, the dynamical equation for SCs can be derived:

2 $$\frac{{{\rm{d}}}^{2}{\phi }_{c}}{{\rm{d}}{\tau }^{2}}+2\frac{{\rm{d}}{\phi }_{c}}{{\rm{d}}\tau }-\frac{F}{M}=0$$

The above equation is a typical forced oscillation equation. The effective kinetic mass of soliton is derived as *M* = *E* (given by momentum analysis, see SI for details), where *E* is the optical pulse energy. Based on Eq. (2), we calculate the evolution trace of the SC angular position. As shown in Fig. 5b, where three adjacent lattice sites of a 10-SC oscillate in phase. The amplitude of the oscillation is calculated as ~0.001 rad (~4% of the soliton width).

The forced oscillation also induces the periodical variation of CW, and causes the SC power to oscillate. The measured normalized soliton power trace shows the periodic modulation (middle panel of Fig. 5d), with a modulation depth of ~10%. The modulation frequency is 69 MHz, which is verified by electrical measurement, see right panel of Fig. 5d, where the second harmonic of 138 MHz is also detected. It should be noted that this power oscillation is fundamentally distinct from the power breathing in the breather soliton, as its optical spectrum maintains the stable *sech*^2^ envelope instead of the typical triangle envelope of breather^31,32^. These experimental observations are further validated by DLLE-based numerical simulations (Fig. 5e). The forced oscillation corresponds to sidebands generation around the comb line in the forms of cross-phase modulation (XPM) and subsequent FWM comb lines caused by XPM and SC comb, the experimental and numerical characterization of XPM comb are included in SI. The forced oscillation of the SC may be eliminated once the control beam is switched off. However, the process of switching off the control beam while maintaining the soliton states is complicated by the thermal effect in the microresonator. Further investigation to reduce the thermal effect is required to eliminate the control beam after the formation of SC states, and this is possible through optimizing the fabrication process of microresonators^52,53^.

### Soliton oscillation frequency and repetition rate tuning

**Fig. 6:**
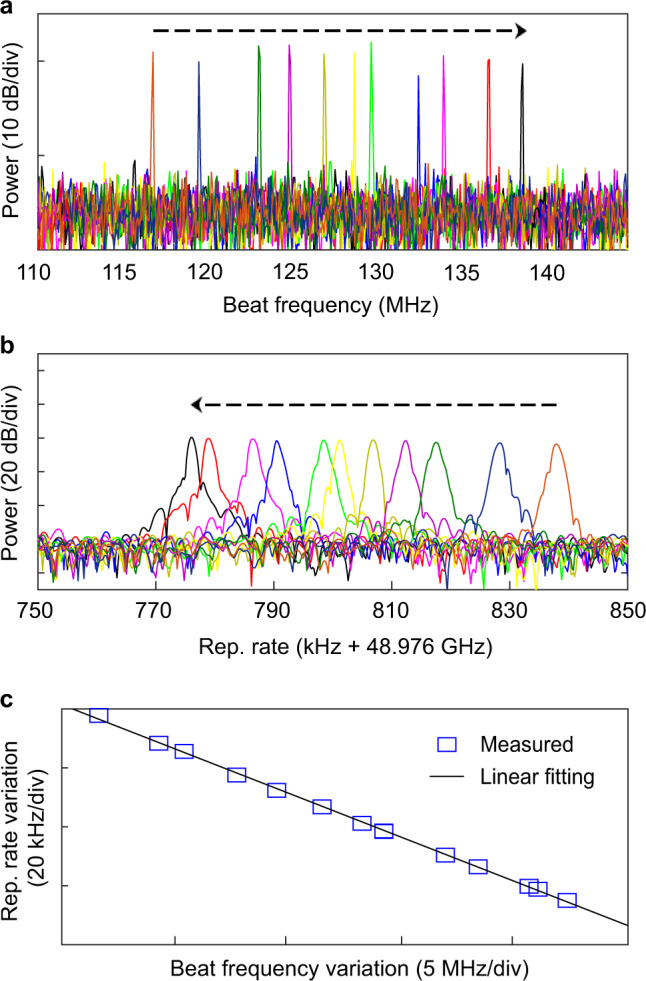
Soliton repetition rate tuning. **a**, **b** The measured beat frequency Δ*f* (i.e., soliton oscillation frequency) and repetition rate of a single soliton as the frequency of the control laser is tuned. **c** The repetition rate of the single soliton is approximately linearly decreasing with the increasing beat frequency, providing a way for realizing oscillation frequency and repetition rate tuning.

Furthermore, due to the photothermal effect, the oscillation frequency and the repetition rate of synthesized SC can be tuned by adjusting the frequency of the control light. As the single-soliton state has an electronic-detectable repetition rate, we study the tuning process of a single-soliton state as an example. Experimentally, the oscillation frequency and repetition rate are directly measured using two high-speed ESAs to monitor the tuning process. The frequency of the control laser is tuned finely by gradually changing the driven current of ECDL. For each driven current of the control light, we record both the oscillation frequency and the repetition rate of the single-soliton comb, as shown in Fig. 6a, b, respectively.

In our measurement, the oscillation frequency of the soliton microcomb is tuned over 20.6 MHz (Fig. 6a), and the repetition rate changes about 60 kHz (Fig. 6b). The soliton repetition rate is linearly changed along with the oscillation frequency (Fig. 6c). It provides a way to finely tune soliton repetition rate and oscillation frequency.

## Discussion

Here, we explore the SC synthesis on demand and the forced oscillation process of the SC. The artificial background potential field is potentially reconfigurable in real-time, provides a platform for studying the cavity Kerr soliton dynamics. The demonstrated reconfigurable SC microcombs in a monolithic integrated microresontors with the repetition rate covering broad microwave bands (including V band, W band, and G band) and THz band, has great potential in applications such as satellite communications^54^, photonic radar^55^, radio-frequency filter^56,57^, and THz technologies. The proposed approach is easy to implement, and therefore applicable to most materials and different cavity configurations.

## Methods

### Controllable artificial background field

A pre-designed background is proposed to change the soliton generation dynamics and regulate the soliton distribution and quantity. Operationally, this can be realized by adding an additional control light that is *N* × FSR from the primary pump light. This leads to the formation of controllable artificial background field expressed as: $\widetilde{{E}_{b}}={E}_{p}{e}^{-i{\omega }_{p}t}{e}^{i{\mu }_{p}{\phi }_{l}}+{E}_{c}{e}^{-i{\omega }_{c}t}{e}^{i{\mu }_{c}{\phi }_{l}}$, where *E*_*p*,*c*_ and *ω*_*p*,*c*_ represent the electric field amplitude and angular frequency of the pump (control) laser, respectively. *ϕ*_*l*_ is the polar angle in lab coordinate. In the rotating angular coordinate *ϕ* = *ϕ*_*l*_ − *D*_1_*t* where *D*_1_ = 2*π* × *FSR*, the background field can be rewritten as $\widetilde{{E}_{b}}=({E}_{p}+{E}_{c}{e}^{-i{{\Delta }}\omega t}{e}^{i\eta \phi }){e}^{-i{\omega }_{p}t}{e}^{i{\mu }_{p}{\phi }_{l}}$, which is *N*-periodically modulated (*N* = *η* = *μ*_*c*_ − *μ*_*p*_). Δ*ω* = *ω*_*c*_ − *ω*_*p*_ − *ηD*_1_ is the frequency mismatch. Δ*ω*/*η* is the traveling speed of the background field, which could be adjusted by tuning the frequency (detuning) of the control light (detailed derivation see Supplementary information). The modulated background field redistributes the refractive index of the cavity in a periodic fashion (Fig. 1c), and regularizes the soliton formation probability around the cavity. Figure 1d shows a synthesized SC where the solitons are evenly distributed with a smooth spectral envelope. Note that if there is a relative motion between the SC and the field, i.e., Δ*ω* is detuned from zero, the SC would periodically oscillate in the temporal domain, which corresponds to modulation sidebands generation in the frequency domain as shown in the inset of Fig. 1d.

## Supplementary information

Supplementary Information

## Data Availability

The data that support the findings of this study are available from the corresponding author upon reasonable request.

## Code availability

The code for the data analysis in this article is available from the corresponding author upon reasonable request.
