# Supplementary material for: Synthesized soliton crystals
Source: Nat Commun. 2021 May 26;12:3179. doi: 10.1038/s41467-021-23172-2 (PMC8154952; doi:10.1038/s41467-021-23172-2)
Supplement: Supplementary file 1 — Supplementary Information [file 41467_2021_23172_MOESM1_ESM.pdf]

Supplementary Information - Synthesized soliton crystals

Zhizhou Lu[1], Hao-Jing Chen[2], Weiqiang Wang[1,3], Lu Yao[2],
Yang Wang[1,3], Yan Yu[2], B. E. Little[1,3], S. T. Chu[4], Qihuang Gong[2,5,6],
Wei Zhao[1,3], Xu Yi[7,8], Yun-Feng Xiao[2,5,6*], and Wenfu Zhang[1,3*]

[1]State Key Laboratory of Transient Optics and Photonics, Xi'an Institute of Optics and Precision Mechanics, Chinese Academy of Sciences, Xi'an 710119, China.
[2]State Key Laboratory for Mesoscopic Physics and Frontiers Science Center for Nano-optoelectronics, School of Physics, Peking University, 100871 Beijing, China.
[3]University of Chinese Academy of Sciences, Beijing 100049, China.
[4]Department of Physics and Materials Science, City University of Hong Kong.
[5]Collaborative Innovation Center of Quantum Matter, Beijing 100871, China.
[6]Collaborative Innovation Center of Extreme Optics, Shanxi University, 030006 Taiyuan, China.
[7]Department of Electrical and Computer Engineering, University of Virginia, Charlottesville, VA 22904, USA.
[8]Department of Physics, University of Virginia, Charlottesville, VA 22904, USA.
These authors contributed equally: Zhizhou Lu, Hao-Jing Chen, Weiqiang Wang, Lu Yao
[*]email: wfuzhang@opt.ac.cn; yfxiao@pku.edu.cn

April 15, 2021

This Supplementary Information is organized as follows. In Section 1, we summarize the device characterization, including the measurements of loaded quality factor, waveguide power reflection parameter and integrated dispersion, these parameters are further used in the numerical simulation. In Section 2, we summarize the theory of synthesized soliton crystal, including the theoretical analysis of dichromatic-pump field, dichromatic-pump Lugiato-Lefever model,

basic pulse dynamics, synthesized potential field and oscillated dynamics of soliton crystals. In Section 3, we provide more details of the experiment, including the four representative SC intracavity power traces all showing direct transition from modulated Turing pattern (TP) and the control-laser based XPM comb generation.

1 Device characterization

The four-port integrated microcavity (radius = 592.1 µm, FSR = $\sim$ 48.9 GHz) is based on CMOS-compatible low loss ($\sim$ 0.06 dB/cm at C band), high-index (n=1.6) doped silica platform (*1*) . The waveguides are surrounded by SiO_2 cladding. During the fabrication process, the core film is deposited using chemical vapor deposition, then the device patterns are printed in photoresist using in-line stepper and etched by reactive ion etching (*2*) . To improve the light on-chip coupling efficiency, a mode transformer (MT) structure is added to the four ports of the device, which allows optimized coupling loss of $\sim$ 2.5 dB per facet (*3*). The loaded Q of our device is $\sim$ 2.63 million, as shown in Supplementary Figure 1(a), where the transmission curve from the through port is measured using a high-speed oscilloscope. A Mach-Zehnder interferometer (MZI) with the period of $\sim$40 MHz is used to measure the full width at half maximum (FWHM) of the Lorentz fitted transmission curve. To measure the back reflection parameter, we inject a light into port 1 (see inset of Supplementary Figure 1(b)), and collect the power from port 3 (drop) and 1 (add), respectively. The results and fitting are shown in Supplementary Figure 1(b), where the power is normalized to the peak of the fitting curve for drop port, the inset indicates that the power reflected from the drop port is $\sim 3.27\%$. Supplementary Figure 1(c) shows the measured and fitted integrated dispersion of the utilized resonator, from which we extract the FSR and second-order dispersion parameters at mode 0 (1560.2 nm): FSR = 48.98 GHz, D_2 = $2\pi \times$ 126.3 kHz. These parameters are further used in numerical simulation.

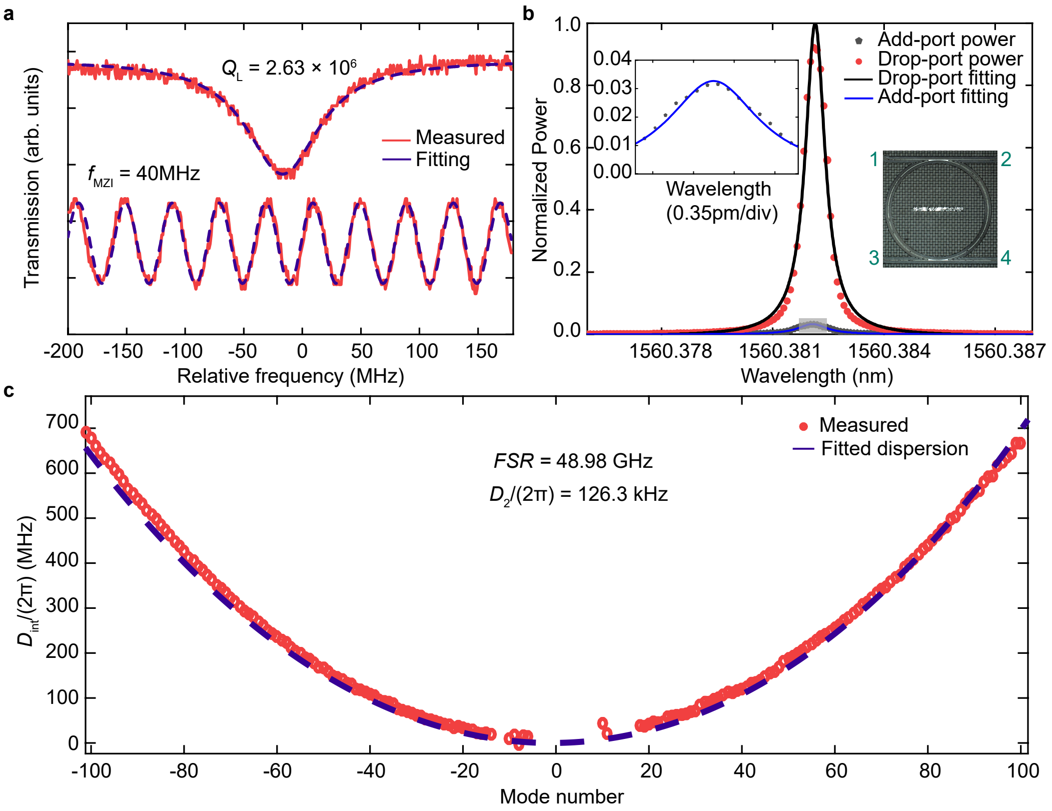

Supplementary Figure 1. Characterization of the microcavity. (a) Measured and fitting transmission curve from the through port of the cavity, the loaded Q factor is 2.63 million, which is confirmed with an MZI featuring 40MHz period. (b) Simultaneously measured and fitting power from add- and drop- port, showing $\sim$ 3.27% of power reflection. (c) Measured and fitted D_{int} versus mode number.

2 Theory of synthesized soliton crystal

For simplicity, we only consider the part of the control light which co-propagrates with the pump light. The pump light field can be expressed as $\widetilde{E}_p = E_p e^{-i\omega_p t} e^{i\mu_p \phi_l}$, while the control light field can be expressed as $\widetilde{E}_c = E_c e^{-i\omega_c t} e^{i\mu_c \phi_l}$. E_p (E_c) is the complex amplitude of pump (control) light, ω_p (ω_c) is the angular frequency of pump (control) light, μ_p (μ_c) is the mode number of pump (control) mode, ϕ_l is the polar angle in laboratory coordinate.

2.1 Dichromatic-pump field

The total electrical field injected into the microcavity is the sum of the pump field and the control field, which can be expressed as:

$$\begin{aligned}
\widetilde{E_{\text{in}}} &= E_p e^{-i\omega_p t} e^{i\mu_p \phi_l} + E_c e^{-i\omega_c t} e^{i\mu_c \phi_l} \\
&= (E_p + E_c e^{-i(\omega_c - \omega_p)t} e^{i(\mu_c - \mu_p)\phi_l}) e^{-i\omega_p t} e^{i\mu_p \phi_l} \\
&= (E_p + E_c e^{-i(\omega_c - \omega_p - (\mu_c - \mu_p)D_1)t} e^{i(\mu_c - \mu_p)(\phi_l - D_1 t)}) e^{-i\omega_p t} e^{i\mu_p \phi_l} \\
&= (E_p + E_c e^{-i\Delta\omega t} e^{i\eta\phi}) e^{-i\omega_p t} e^{i\mu_p \phi_l}
\end{aligned} \tag{1}$$

Here, $\phi = \phi_l - D_1 t$ is the rotating angular coordinate with angular velocity $D_1 = 2\pi \times \text{FSR}$ (*4*). $\eta = \mu_c - \mu_p$ denotes the mode number difference between control light and pump light, and $\Delta\omega = \omega_c - \omega_p - \eta D_1$ is the beat angular frequency or the frequency mismatch. By taking the frequency and mode number of the pump laser as the reference (which eliminates the $e^{-i\omega_p t} e^{i\mu_p \phi_l}$ factor in Eq. 1), the total injected electrical field is reduced to

$$\widetilde{E_{\text{in}}}' = E_p + E_c e^{-i\Delta\omega t} e^{i\eta\phi} \tag{2}$$

which oscillates periodically with a frequency of $\Delta\omega/2\pi$. The term $E_c e^{-i\Delta\omega t} e^{i\eta\phi}$ exhibits the behaviour of a traveling wave, with frequency $\Delta\omega/2\pi$, spatial periodicity η and traveling speed $\Delta\omega/\eta$.

2.2 Dichromatic-pump LLE model

When introducing the additional control light, the LLE can be rewritten as:

$$\frac{\partial A(\phi,t)}{\partial t}=i\frac{D_2}{2}\frac{\partial^2 A}{\partial\phi^2}+ig|A|^2A-\left(i\delta\omega+\frac{\kappa}{2}\right)A+f_p+f_c e^{i(\eta\phi+\varphi_2)}e^{-i\Delta\omega t} \tag{3}$$

where an additional pump term is added according to Eq. 2. Here $A(\phi,t)$ is the slowly varying intracavity field amplitude defined on the rotating angular coordinate $\phi=\phi_l-D_1t$. D_2 is the second-order group velocity dispersion (GVD) term. The input field $f_p=\sqrt{P_p/\hbar\omega_0}$, $f_c=r_{\text{eff}}\sqrt{RP_c/\hbar\omega_0}$, where $R=3.27\%$ is the maximum power reflection rate, and r_{eff} denotes the power coefficient related to the control laser detuning. In the simulation, we link this value to the measured transmission spectrum shown in Supplementary Figure 1(a). φ_2 is the initial phase of control light. $\eta=\mu_c-\mu_p$ denotes the difference of mode number between control light mode and pump light mode. The laser-cavity detuning is defined as $\delta\omega=\omega_0-\omega_p$, where ω_0 and ω_p are the angular frequencies of the pumped resonance and the CW pump light, correspondingly. Total cavity losses are described with the photon decay rate κ. The Kerr nonlinear coefficient g is defined as $g=\hbar\omega_0^2cn_2/n_0^2V_{\text{eff}}$, where n_2 is the nonlinear refractive index, n_0 is the effective group refractive index and V_{eff} is the effective optical mode volume.

The above LLE can be normalized for convenience by taking $t=2\tau/\kappa$ and $A=\sqrt{\kappa/2g}\psi$, where τ is the normalized time, ψ is the normalized optical field:

$$\frac{\partial\psi(\phi,\tau)}{\partial\tau}=i\beta\frac{\partial^2\psi}{\partial\phi^2}+i|\psi|^2\psi-(i\zeta+1)\psi+\tilde{f}_p+\tilde{f}_c e^{i(\eta\phi+\varphi_2)}e^{-i\Delta\tilde{\omega}\tau} \tag{4}$$

Here, $\beta=D_2/\kappa$, $\Delta\tilde{\omega}=2(\omega_c-\omega_p-\eta D_1)/\kappa$, $\zeta=2\delta\omega/\kappa$, $\tilde{f}_p=\sqrt{8g/\kappa^3}f_p$, $\tilde{f}_c=\sqrt{8g/\kappa^3}f_c$. The simulations in the main text are performed by numerically integrates the above equation (Eq. S9) with a split-step Fourier method. We use the measured parameters of the real device from our experiment, which includes: FSR = 48.98 GHz, $D_2/2\pi$ = 126.3 kHz, $\kappa/2\pi$ = 73 MHz. The Raman effect and higher order dispersion terms are neglected.

2.3 Basic pulse dynamics

By introducing the total drive term $\tilde{F} = \tilde{f}_p + \tilde{f}_c e^{i(\eta\phi+\varphi_2)} e^{-i\Delta\tilde{\omega}\tau}$, Eq. 4 can be reduced further as:

$$\frac{\partial\psi(\phi,\tau)}{\partial\tau} = i\beta\frac{\partial^2\psi}{\partial\phi^2} + i|\psi|^2\psi - (i\zeta+1)\psi + \tilde{F} \tag{5}$$

The intracavity field can be Fourier expanded as

$$\psi(\phi,\tau) = \sum_\mu \psi_\mu(\tau) e^{i\mu\phi} \tag{6}$$

where ψ_μ denotes the optical field of the μ-th comb line and $|\psi_\mu|^2$ is the normalized optical energy or photon number of the μ-th mode.

To study the dynamics of a pulse, we can think of it as a particle and study its motion in the rotating angular coordinate. The optical energy is given in Ref. (*5*), since the constant (i.e., speed of light in vacuum) relation between energy and mass could be normalized, here we also link this equation to the mass of the pulse:

$$M \equiv E \equiv \sum_\mu |\psi_\mu|^2 = \frac{1}{2\pi}\int_0^{2\pi} |\psi|^2 d\phi \tag{7}$$

Note that the resonance frequency of the μ-th mode (relative to the pump mode) can be approximated with Taylor expansion

$$\omega_\mu = \omega_0 + D_1\mu + \frac{1}{2}D_2\mu^2 \tag{8}$$

therefore, the angular group velocity of the μ-th mode is

$$\omega_g = \frac{\mathrm{d}\omega_\mu}{\mathrm{d}\mu} = D_1 + D_2\mu \tag{9}$$

In the rotating angular coordinate, the normalized relative angular group velocity is $2D_2\mu/\kappa = 2\beta\mu$. Therefore, the momentum of the pulse can be defined as the momentum summation over

all optical modes

$$P \equiv \sum_{\mu} 2\beta\mu|\psi_\mu|^2 = \frac{\beta}{2\pi}\int_0^{2\pi} \mathrm{d}\phi\psi^* \left(-i\frac{\partial}{\partial\phi}\right)\psi + c.c. = \frac{\beta}{\pi}\int_0^{2\pi} |\psi|^2\frac{\partial\varphi}{\partial\phi}\mathrm{d}\phi \tag{10}$$

where $\varphi(\phi,\tau)$ is the argument of ψ. From Eq. 7 and 10, it is clear that in the spatial-temporal domain, we can define the mass density $\rho(\phi,\tau)$ and the momentum density $J(\phi,\tau)$ as

$$\rho(\phi,\tau) \equiv \frac{1}{2\pi}|\psi|^2 \qquad J(\phi,\tau) \equiv \frac{\beta}{\pi}|\psi|^2\frac{\partial\varphi}{\partial\phi} \tag{11}$$

From Eq. 5 and Eq. 11, we could obtain:

$$\begin{aligned}\frac{\partial\rho}{\partial\tau} &= \frac{1}{2\pi}\psi^*\frac{\partial\psi}{\partial\tau} + c.c.\\ &= \frac{1}{2\pi}\psi^*\left(i\beta\frac{\partial^2\psi}{\partial\phi^2} + i|\psi|^2\psi - (i\zeta+1)\psi + \tilde{F}\right) + c.c.\\ &= \frac{i\beta}{2\pi}\left(\psi^*\frac{\partial^2\psi}{\partial\phi^2} - \psi\frac{\partial^2\psi^*}{\partial\phi^2}\right) - 2\rho + \frac{1}{\pi}\mathrm{Re}(\psi^*\tilde{F})\\ &= \frac{i\beta}{2\pi}\frac{\partial}{\partial\phi}\left(\psi^*\frac{\partial\psi}{\partial\phi} - \psi\frac{\partial\psi^*}{\partial\phi}\right) - 2\rho + \frac{1}{\pi}\mathrm{Re}(\psi^*\tilde{F})\\ &= -\frac{\partial J}{\partial\phi} - 2\rho + \frac{1}{\pi}\mathrm{Re}(\psi^*\tilde{F})\end{aligned} \tag{12}$$

The first term on the right hand side represents the density flow, while the second and third term correspond to dissipation and pump driving respectively. When deleting the second and third term (loss and pump) we have

$$\frac{\partial\rho}{\partial\tau} + \frac{\partial J}{\partial\phi} = 0 \tag{13}$$

which is the one-dimensional mass conservation equation in fluid dynamics. This proves that the definition of mass M and momentum P is reasonable and our dynamic theory is self-consistent. Furthermore, the velocity field can be defined as

$$v(\phi,\tau) \equiv \frac{J(\phi,\tau)}{\rho(\phi,\tau)} = 2\beta\frac{\partial\varphi}{\partial\phi} \tag{14}$$

For a short pulse stably propagating in the microcavity, the velocity (therefore $\partial\varphi/\partial\phi$) around the pulse center can be regarded as a constant, meaning that the optical field of the pulse can be approximated as

$$\psi(\phi,\tau) = |\psi| \exp\left\{ i \left[\varphi(\phi_c) + \left(\frac{\partial\varphi}{\partial\phi}\right)_{\phi=\phi_c} (\phi - \phi_c) \right] \right\} \tag{15}$$

where $\phi_c = \phi_c(\tau)$ is the center position of the pulse which may change over time. Since each part of the pulse is considered to have the same speed, the velocity of the pulse should be expressed as

$$\frac{\mathrm{d}\phi_c}{\mathrm{d}\tau} = v = \frac{P}{M} = 2\beta \frac{\sum_\mu \mu|\psi_\mu|^2}{\sum_\mu |\psi_\mu|^2} \equiv 2\beta\mu_{\mathrm{cen}} \tag{16}$$

where $\mu_{\mathrm{cen}} \equiv \sum_\mu \mu|\psi_\mu|^2 / \sum_\mu |\psi_\mu|^2$ is the spectral center mode number. Therefore we have

$$\frac{\partial\varphi}{\partial\phi} = \frac{v}{2\beta} = \mu_{\mathrm{cen}} \tag{17}$$

$$\psi(\phi,\tau) = |\psi| \exp\left\{ i \left[\varphi(\phi_c) + \mu_{\mathrm{cen}}(\phi - \phi_c) \right] \right\} \tag{18}$$

2.4 Synthesized force field and equation of motion for optical solitons

To study the motion of a pulse, we start by taking the time derivative of Eq. 7 and 10

$$\begin{aligned}
\frac{\mathrm{d}M}{\mathrm{d}\tau} &= \frac{1}{2\pi}\int_0^{2\pi}\psi^*\frac{\partial\psi}{\partial\tau}\mathrm{d}\phi + c.c. \\
&= \frac{1}{2\pi}\int_0^{2\pi}\psi^*\left[i\beta\frac{\partial^2\psi}{\partial\phi^2} + i|\psi|^2\psi - (i\zeta+1)\psi + \tilde{F}\right]\mathrm{d}\phi + c.c. \\
&= \frac{1}{2\pi}\int_0^{2\pi}\psi^*\left[-\psi + \tilde{f}_p + \tilde{f}_c e^{i(\eta\phi+\varphi_2)}e^{-i\Delta\tilde{\omega}\tau}\right]\mathrm{d}\phi + c.c. \\
&= -2(M - M_0) + \frac{1}{2\pi}\int_0^{2\pi}\left[\psi^*\tilde{f}_c e^{i(\eta\phi+\varphi_2)}e^{-i\Delta\tilde{\omega}\tau}\mathrm{d}\phi + c.c.\right] \qquad (19)\\
\frac{\mathrm{d}P}{\mathrm{d}\tau} &= \frac{\beta}{2\pi}\int_0^{2\pi}\mathrm{d}\phi\left[\frac{\partial\psi^*}{\partial\tau}\left(-i\frac{\partial}{\partial\phi}\right)\psi + \psi^*\left(-i\frac{\partial^2}{\partial\phi\partial\tau}\right)\psi\right] + c.c. \\
&= \frac{\beta}{\pi}\int_0^{2\pi}\mathrm{d}\phi\frac{\partial\psi^*}{\partial\tau}\left(-i\frac{\partial}{\partial\phi}\right)\psi + c.c. \\
&= \frac{\beta}{\pi}\int_0^{2\pi}\mathrm{d}\phi\left[-i\beta\frac{\partial^2\psi^*}{\partial\phi^2} - i|\psi|^2\psi^* - (-i\zeta+1)\psi^* + \tilde{F}^*\right]\left(-i\frac{\partial}{\partial\phi}\right)\psi + c.c. \\
&= -2P + \frac{\beta}{\pi}\int_0^{2\pi}\mathrm{d}\phi\left[\psi^*\left(-i\frac{\partial}{\partial\phi}\right)\tilde{F} + c.c.\right] \\
&= -2P + F \qquad (20)
\end{aligned}$$

where $M_0 = \int_0^{2\pi}\psi^*\tilde{f}_p d\phi/4\pi + c.c.$ and $F = (\beta/\pi)\int_0^{2\pi}\mathrm{d}\phi\psi^*(-i\partial/\partial\phi)\tilde{F} + c.c.$. The first term of Eq. 20 shows the dissipation, while the second term denotes the driving force applied by the control laser.

When there is a single soliton circulating the microcavity, the optical field can be approximately described as

$$\psi(\phi,\tau) = \psi_b + \psi_s = \psi_b + B_s\mathrm{sech}\left(\frac{\phi-\phi_c(\tau)}{\phi_\tau}\right)\exp\left\{i[\mu_{\mathrm{cen}}(\phi-\phi_c(\tau)) + \varphi_s]\right\} \qquad (21)$$

where $\psi_b = \tilde{f}_p/[1 + i(\zeta - |\psi_b|^2)]$ is the CW background and ψ_s is the characteristic of optical solitons. The parameters are $B_s = \sqrt{2\zeta}$, $\phi_\tau = \sqrt{\beta/\zeta}$, and $\varphi_s = \arccos(\sqrt{8\zeta}/\pi\tilde{f}_p)$. Around the pulse center, the sech term ψ_s dominates, while away from the pulse center the CW background dominates. The velocity of the homogeneous CW background is strictly zero (locked

by the pump laser), meaning that it does not move together with the sech part. Mathematically speaking, it is easy to verify that

$$
\begin{aligned}
P=& \frac{\beta}{2\pi}\int_0^{2\pi} \mathrm{d}\phi\psi^* \left(-i\frac{\partial}{\partial\phi}\right)\psi + c.c. \\
=& \frac{\beta}{2\pi}\int_0^{2\pi} \mathrm{d}\phi\psi_s^* \left(-i\frac{\partial}{\partial\phi}\right)\psi_s + c.c. \qquad (22)\\
F=& \frac{\beta}{\pi}\int_0^{2\pi} \mathrm{d}\phi\psi^* \left(-i\frac{\partial}{\partial\phi}\right)\tilde{F} + c.c. \\
=& \frac{\beta}{\pi}\int_0^{2\pi} \mathrm{d}\phi\psi_s^* \left(-i\frac{\partial}{\partial\phi}\right)\tilde{F} + c.c. \qquad (23)
\end{aligned}
$$

meaning that the stationary CW background has no contribution to either the total momentum or the total force applied to the intracavity field. In short, ψ_s travels independently on top of the stationary CW background. Therefore, when studying the soliton motion, one should ignore ψ_b and focus entirely on ψ_s (*5*). For convenience, from now on we omit ψ_b and take $\psi = \psi_s$. We have

$$
\begin{aligned}
M_0=& \frac{1}{4\pi}\int_0^{2\pi} \psi^* \tilde{f}_p d\phi + c.c. \\
=& \frac{1}{4}B_s\tilde{f}_p\phi_\tau \mathrm{sech}\left(\frac{\pi\mu_{\mathrm{cen}}\phi_\tau}{2}\right)\mathrm{e}^{-\mathrm{i}\varphi_s} + \mathrm{c.c.} \\
\approx& \frac{1}{2}B_s\phi_\tau\tilde{f}_p\cos\varphi_s \\
=& \frac{2}{\pi}\sqrt{\beta\zeta} \qquad (24)
\end{aligned}
$$

Note that when $\tilde{f}_c$ is small, we have $|\mu_{\mathrm{cen}}| \ll 1$, $|M - M_0| \ll M_0$, and $|P| \ll M_0$.

According to Eq. 20, the driving force applied to the soliton by the control laser can be

written as

$$
\begin{aligned}
F(\phi_c) &= \frac{\beta}{\pi}\int_0^{2\pi} \mathrm{d}\phi \left[\psi^* \left(-i\frac{\partial}{\partial\phi}\right)\tilde{F} + c.c.\right] \\
&= \frac{\beta}{\pi} B_s e^{-i\varphi_s} \int_0^{2\pi} \mathrm{d}\phi \,\mathrm{sech}\left(\frac{\phi-\phi_c}{\phi_\tau}\right) e^{-i\mu_{\mathrm{cen}}(\phi-\phi_c)} \left(-i\frac{\partial}{\partial\phi}\right) \tilde{f}_c e^{i(\eta\phi+\varphi_2)} e^{-i\Delta\widetilde{\omega}\tau} + c.c. \\
&= \frac{\beta}{\pi}\eta B_s \tilde{f}_c e^{i(\eta\phi_c+\varphi_2-\varphi_s-\Delta\widetilde{\omega}\tau)} \int_0^{2\pi} \mathrm{d}\phi \,\mathrm{sech}\left(\frac{\phi-\phi_c}{\phi_\tau}\right) e^{i(\eta-\mu_{\mathrm{cen}})(\phi-\phi_c)} + c.c. \\
&= \beta\eta B_s \phi_\tau \tilde{f}_c \mathrm{sech}\left[\frac{\pi(\eta-\mu_{\mathrm{cen}})\phi_\tau}{2}\right] e^{i(\eta\phi_c+\varphi_2-\varphi_s-\Delta\widetilde{\omega}\tau)} + c.c. \\
&\approx 2\beta\eta B_s \phi_\tau \tilde{f}_c \,\mathrm{sech}\left(\frac{\pi\eta\phi_\tau}{2}\right)\cos(\eta\phi_c+\varphi_2-\varphi_s-\Delta\widetilde{\omega}\tau) \\
&= F_0 \cos(\eta\phi_c+\varphi_2-\varphi_s-\Delta\widetilde{\omega}\tau)
\end{aligned} \tag{25}
$$

where $F_0 \equiv 2\beta\eta B_s\phi_\tau\tilde{f}_c\,\mathrm{sech}(\pi\eta\phi_\tau/2) = (2\beta)^{3/2}\eta\tilde{f}_c\mathrm{sech}(\pi\eta\sqrt{\beta/\zeta}/2)$. Therefore the potential field can be written as

$$
V(\phi_c) = -\int F \mathrm{d}\phi_c = -\frac{F_0}{\eta}\sin(\eta\phi_c+\varphi_2-\varphi_s-\Delta\widetilde{\omega}\tau) \tag{26}
$$

which behaves as a traveling wave with velocity $\Delta\widetilde{\omega}/\eta$ in the rotating angular coordinate. Since $P = Mv = M\mathrm{d}\phi_c/\mathrm{d}\tau$, together with Eq. 19 and 20, we obtain the following dynamic equations

$$
\frac{\mathrm{d}M}{\mathrm{d}\tau} + 2(M-M_0) = \frac{F}{2\beta\eta} \tag{27}
$$

$$
\frac{\mathrm{d}}{\mathrm{d}\tau}\left(M\frac{\mathrm{d}\phi_c}{\mathrm{d}\tau}\right) + 2M\frac{\mathrm{d}\phi_c}{\mathrm{d}\tau} = F \tag{28}
$$

Since F is small, $|\mathrm{d}M/\mathrm{d}\tau| \ll M$, Eq. 28 can be approximated as (to the first order of F)

$$
\frac{\mathrm{d}^2\phi_c}{\mathrm{d}\tau^2} + 2\frac{\mathrm{d}\phi_c}{\mathrm{d}\tau} - \frac{F}{M} = 0 \tag{29}
$$

which is the Eq. 2 in the main text.

2.5 Motion of soliton crystal

2.5.1 When the frequency mismatch $\Delta\widetilde{\omega}$ is small: trapped by the potential field

When $\Delta\widetilde{\omega}$ is small, soliton pulses move together with the potential field (*4*), meaning that

$$\frac{\mathrm{d}\phi_c}{\mathrm{d}\tau} = \frac{\Delta\widetilde{\omega}}{\eta} \tag{30}$$

Together with Eq. 29 we obtain

$$F = 2M\frac{\mathrm{d}\phi_c}{\mathrm{d}\tau} \approx 2M_0\frac{\Delta\widetilde{\omega}}{\eta} \tag{31}$$

Therefore together with Eq. 25, the equilibrium position of the soliton is

$$\begin{aligned}\phi_{ce}(\tau) &= \frac{1}{\eta}\left[\pm\arccos\left(\frac{F}{F_0}\right) + 2k\pi + \Delta\widetilde{\omega}\tau + \varphi_s - \varphi_2\right] \\ &= \frac{1}{\eta}\left[\pm\arccos\left(\frac{2M_0\Delta\widetilde{\omega}}{\eta F_0}\right) + 2k\pi + \Delta\widetilde{\omega}\tau + \varphi_s - \varphi_2\right]\end{aligned} \tag{32}$$

where k is an integer. As for the $\pm$ sign which corresponds to two sets of equilibrium positions, only the one set with $(\mathrm{d}F/\mathrm{d}\phi_c)_{\phi_c=\phi_{ce}} < 0$ is stable. This set of stable equilibrium positions exists only when

$$|\Delta\widetilde{\omega}| < \Delta\widetilde{\omega}_{\max} = \eta F_0/2M_0 = \pi\beta\eta^2\tilde{f}_c\mathrm{sech}(\pi\eta\sqrt{\beta/\zeta}/2)/\sqrt{2\zeta} \tag{33}$$

where $\Delta\widetilde{\omega}_{\max} \sim 0.01$ is the critical value. It is obvious that the stable equilibrium positions are equally spaced by $2\pi/\eta$, which stabilizes the soliton crystal generated during the resonance scanning process.

2.5.2 When the frequency mismatch $\Delta\widetilde{\omega}$ is large: forced oscillation

When $\Delta\widetilde{\omega} > \Delta\widetilde{\omega}_{\max}$, solitons can no longer move synchronously with the potential field, which is the case in the experiment. Mathematically speaking, when $\Delta\widetilde{\omega} \gg \Delta\widetilde{\omega}_{\max}$, to the zeroth

order of F_0, the soliton center position $\phi_c \approx \phi_{c0}$ and mass $M \approx M_0$ remain constant; To the first order of F_0,

$$\phi_c \approx \phi_{c0} + \phi_{c1}\cos(\Delta\widetilde{\omega}\tau + \delta_{\phi 1}) \tag{34}$$

$$M \approx M_0 + M_1\cos(\Delta\widetilde{\omega}\tau + \delta_{M1}) \tag{35}$$

where $\phi_{c1}, M_1 \propto F_0^1$; To the second order of F_0,

$$M \approx M_0 + M_1\cos(\Delta\widetilde{\omega}\tau + \delta_{M1}) + M_{21} + M_{22}\cos(2\Delta\widetilde{\omega}\tau + \delta_{M2}) \tag{36}$$

where $M_{21}, M_{22} \propto F_0^2$; And so on to higher orders. (Here for the amplitude of each frequency oscillation component, only the lowest order of F_0 is kept.) Now we are going to illustrate these mathematical results by the step iterative method.

By replacing ϕ_c with its zeroth order approximation ϕ_{c0} in the expression of F (Eq. 25), Eq. 27 and 29 can be simplified to

$$\frac{\mathrm{d}M}{\mathrm{d}\tau} + 2(M - M_0) = \frac{F_0}{2\beta\eta}\cos(\Delta\widetilde{\omega}\tau') \tag{37}$$

$$\frac{\mathrm{d}^2\phi_c}{\mathrm{d}\tau'^2} + 2\frac{\mathrm{d}\phi_c}{\mathrm{d}\tau'} = \frac{F_0}{M_0}\cos(\Delta\widetilde{\omega}\tau') \tag{38}$$

where $\Delta\widetilde{\omega}\tau' = \Delta\widetilde{\omega}\tau - \eta\phi_{c0} - \varphi_2 + \varphi_s$. The steady-state solution is

$$M = M_0 + \frac{F_0}{2\beta\eta}\frac{1}{\sqrt{\Delta\widetilde{\omega}^2 + 4}}\cos(\Delta\widetilde{\omega}\tau' - \delta_1) \tag{39}$$

$$\phi_c = \phi_{c0} + \frac{F_0}{M_0}\frac{1}{\Delta\widetilde{\omega}\sqrt{\Delta\widetilde{\omega}^2 + 4}}\sin(\Delta\widetilde{\omega}\tau' - \delta_1) \tag{40}$$

where $\delta_1 = \arctan(\Delta\widetilde{\omega}/2)$. These results are the first order approximate solution which verify the aforementioned Eq. 34 and 35. To obtain the second order approximate solution, we replace ϕ_c with its first order approximation in the expression of F and obtain

$$\frac{\mathrm{d}M}{\mathrm{d}\tau} + 2(M - M_0) = \frac{F_0}{2\beta\eta}\cos[\Delta\widetilde{\omega}\tau' - \eta(\phi_c - \phi_{c0})] \tag{41}$$

$$\frac{\mathrm{d}P}{\mathrm{d}\tau} + 2P = F_0\cos[\Delta\widetilde{\omega}\tau' - \eta(\phi_c - \phi_{c0})] \tag{42}$$

The cosinoidal term can be reduced to

$$\begin{aligned}
&\cos[\Delta\widetilde{\omega}\tau' - \eta(\phi_c - \phi_{c0})] \\
=&\mathrm{Re}\{\exp(\mathrm{i}\Delta\widetilde{\omega}\tau')\exp[-\mathrm{i}\eta(\phi_\mathrm{c} - \phi_\mathrm{c0})]\} \\
=&\mathrm{Re}\left\{\exp(\mathrm{i}\Delta\widetilde{\omega}\tau')\exp\left[-\mathrm{i}\frac{\eta\mathrm{F}_0}{\mathrm{M}_0}\frac{1}{\Delta\widetilde{\omega}\sqrt{\Delta\widetilde{\omega}^2+4}}\sin(\Delta\widetilde{\omega}\tau' - \delta_1)\right]\right\} \\
=&\mathrm{Re}\left\{\exp(\mathrm{i}\Delta\widetilde{\omega}\tau')\exp\left[-\mathrm{iz}\sin(\Delta\widetilde{\omega}\tau' - \delta_1)\right]\right\} \\
=&\mathrm{Re}\left\{\exp(\mathrm{i}\Delta\widetilde{\omega}\tau')[\mathrm{J}_0(\mathrm{z}) - 2\mathrm{i}\mathrm{J}_1(\mathrm{z})\sin(\Delta\widetilde{\omega}\tau' - \delta_1) + \mathrm{o}(\mathrm{z})]\right\} \\
=&J_0(z)\cos(\Delta\widetilde{\omega}\tau') + 2J_1(z)\sin(\Delta\widetilde{\omega}\tau' - \delta_1)\sin(\Delta\widetilde{\omega}\tau') + o(z) \\
=&J_0(z)\cos(\Delta\widetilde{\omega}\tau') + J_1(z)[\cos\delta_1 - \cos(2\Delta\widetilde{\omega}\tau' - \delta_1)] + o(z)
\end{aligned} \tag{43}$$

where we use the Jacobi–Anger expansion

$$e^{\pm iz\sin\theta} = J_0(z) + 2\sum_{n=1}^{\infty} J_{2n}(z)\cos(2n\theta) \pm 2i\sum_{n=0}^{\infty} J_{2n+1}(z)\sin((2n+1)\theta) \tag{44}$$

and $z = \eta F_0/M_0\Delta\widetilde{\omega}\sqrt{\Delta\widetilde{\omega}^2+4}$. Since $z \ll 1$, we have $J_0(z) = 1 + o(z)$, $J_1(z) = z/2 + o(z^2)$.

To the second order of F_0, Eq. 41 and 42 are reduced to

$$\frac{\mathrm{d}M}{\mathrm{d}\tau} + 2(M - M_0) = \frac{F_0}{2\beta\eta}\cos(\Delta\widetilde{\omega}\tau') + \frac{F_0^2}{2\beta M_0}\frac{1}{\Delta\widetilde{\omega}(\Delta\widetilde{\omega}^2+4)} - \frac{F_0^2}{4\beta M_0}\frac{1}{\Delta\widetilde{\omega}\sqrt{\Delta\widetilde{\omega}^2+4}}\cos(2\Delta\widetilde{\omega}\tau' - \delta_1) \tag{45}$$

$$\frac{\mathrm{d}P}{\mathrm{d}\tau} + 2P = F_0\cos(\Delta\widetilde{\omega}\tau') + \frac{\eta F_0^2}{M_0}\frac{1}{\Delta\widetilde{\omega}(\Delta\widetilde{\omega}^2+4)} - \frac{\eta F_0^2}{2M_0}\frac{1}{\Delta\widetilde{\omega}\sqrt{\Delta\widetilde{\omega}^2+4}}\cos(2\Delta\widetilde{\omega}\tau' - \delta_1) \tag{46}$$

Therefore we have

$$\begin{aligned}
M=& M_0 + \frac{F_0^2}{4\beta M_0}\frac{1}{\Delta\widetilde{\omega}(\Delta\widetilde{\omega}^2+4)} + \frac{F_0}{2\beta\eta}\frac{1}{\sqrt{\Delta\widetilde{\omega}^2+4}}\cos(\Delta\widetilde{\omega}\tau' - \delta_1) \\
&-\frac{F_0^2}{8\beta M_0}\frac{1}{\sqrt{(\Delta\widetilde{\omega}^2+4)(\Delta\widetilde{\omega}^2+1)}}\cos(2\Delta\widetilde{\omega}\tau' - \delta_1 - \delta_2) + ...
\end{aligned} \tag{47}$$

$$\begin{aligned}
P=& \frac{\eta F_0^2}{2M_0}\frac{1}{\Delta\widetilde{\omega}(\Delta\widetilde{\omega}^2+4)} + F_0\frac{1}{\sqrt{\Delta\widetilde{\omega}^2+4}}\cos(\Delta\widetilde{\omega}\tau' - \delta_1) \\
&-\frac{\eta F_0^2}{4M_0}\frac{1}{\sqrt{(\Delta\widetilde{\omega}^2+4)(\Delta\widetilde{\omega}^2+1)}}\cos(2\Delta\widetilde{\omega}\tau' - \delta_1 - \delta_2) + ...
\end{aligned} \tag{48}$$

where $\delta_2 = \arctan \Delta\widetilde{\omega}$. For the soliton crystal state consisting of η equally spaced pulses, the phase of oscillation is the same for all soliton pulses, meaning that they move synchronously with each other. Therefore, the soliton crystal state is maintained. The above calculations also indicate that the oscillation frequencies are the integer multiples of $\Delta\widetilde{\omega}$, which is in good agreement with both the experiment (Fig. 5d main text) and the simulation (Fig 5e main text) results.

3 Additional measurements

3.1 High Repeatability of SC generation

Benefitting from the periodically modulated background field, SC states could be deterministically formed from TP stage. Through optimizing the pump conditions (polarization, power and frequency), SCs can be synthesized with very high repeatability. We experimentally repeat the SC generation by 20 times for the soliton number N ranging from 1 to 32. For N>10, the synthesized SC states are reached in all trials. For N<10, the success rates are still over 50% (decrease with decreasing N). Supplementary Figure 2 shows four representative intracavity power traces during SC formation process for 2-, 8-, 10- and 20-SC states, each one is overlaid by 5 consecutive attempts.

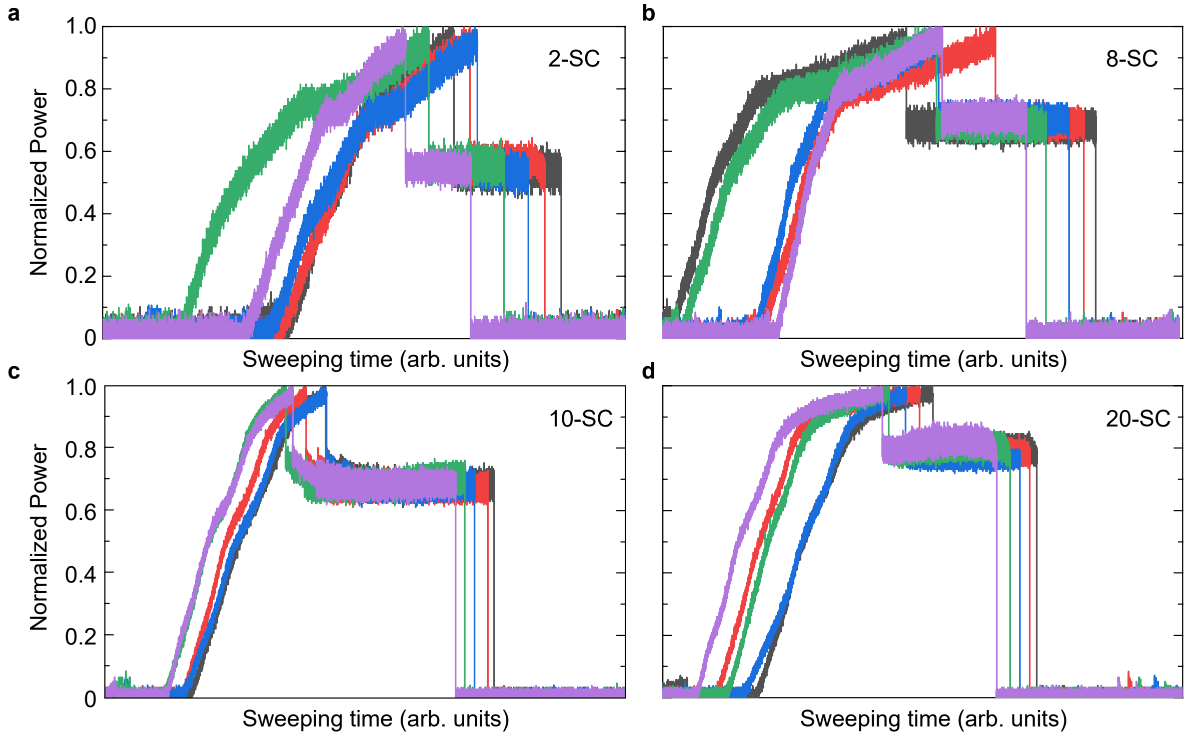

Supplementary Figure 2. Four representative SC evolution power trace. Intracavity power evolution trace for (a) 2-SC, (b) 8-SC, (c) 10-SC and (d) 20-PSC, each one is overlaid by 5 consecutive attempts.

3.2 Cross-phase modulation (XPM) comb

Once SC is synthesized, the index of the microcavity will be periodically modulated which acts on the control light to form an XPM comb. Thus, a secondary comb could be auto-formed which has the same frequency spacing with the synthesized SC. In a recent work, XPM comb has been demonstrated using orthogonally polarized dual-pump scheme, where the weaker TE seed is modulated by the stronger TM soliton comb, thus the XPM comb could be observed below the threshold of the TE comb generation (*6*). For our configuration, the pump and control lasers belong to the same polarization mode family, the modulation and XPM comb are expected to be more obvious due to the larger modulation index (*7*). However, since the XPM comb and SC reside in the same resonances, it is difficult to directly distinguish them using an ordinary OSA. Here, we select a control laser which is about 50-FSR away from the pump laser, the XPM comb coverage spreads out of the soliton spectral range (e.g. the $sech^2$ fitting envelope) and can be directly observed using an OSA. Supplementary Figure 3 shows the corresponding experimental and numerical spectra, where the comb components beyond the fitting curve are XPM comb spectra. The green trace in Supplementary Figure 3(b) shows the simulated XPM comb, which agrees well with the experimental observation shown in Supplementary Figure 3(a) and helps us better resolve the specific comb lines. Note that for simplicity, the group velocity mismatch (GVM) is omitted here. In fact, the GVM only affects the relative power of the two wings (indicated by the black arrows in Supplemetary Figure 3(b)) of the XPM comb spectrum (*7*). The temporal traces of soliton comb and XPM comb are shown in Supplementary Figure 3(c), where the soliton and XPM pulse travel together with a relative velocity compared with the lattice traps. It should be noted that recent studies have shown that the XPM comb could be used for broadening the spectral range (*8, 9*).

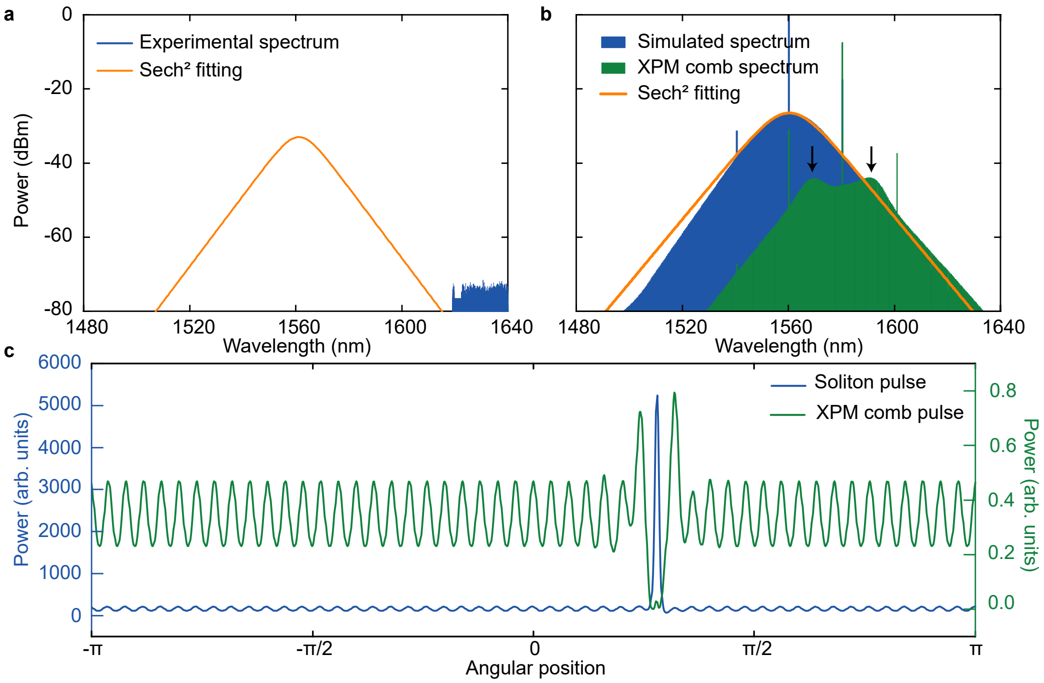

Supplementary Figure 3. Observation of XPM comb by setting the control laser 50 FSR away from the pump.(a) Experimental spectrum and $sech^2$ fitting. (b) Simulated spectrum and $sech^2$ fitting. The XPM comb spectrum is highlighted by green color. (c) Calculated temporal field for soliton and XPM comb.

References

1. Wang, W. *et al.* Dual-pump Kerr micro-cavity optical frequency comb with varying fsr spacing. *Scientific Reports* **6**, 28501–28501 (2016).

2. Razzari, L. *et al.* CMOS-compatible integrated optical hyper-parametric oscillator. *Nat. Photon.* **4**, 41–45 (2010).

3. Wang, W. *et al.* Robust soliton crystals in a thermally controlled microresonator. *Optics Letters* **43**, 2002–2005 (2018).

4. Taheri, H., Matsko, A. B. & Maleki, L. Optical lattice trap for Kerr solitons. *The European Physical Journal D* **71**, 153 (2017).

5. Yi, X. *et al.* Single-mode dispersive waves and soliton microcomb dynamics. *Nat. Commun.* **8**, 14869 (2017).

6. Bao, C. *et al.* Orthogonally polarized frequency comb generation from a Kerr comb via cross-phase modulation. *Optics Letters* **44**, 1472–1475 (2019).

7. Agrawal, G. P. *Nonlinear fiber optics* (Academic press, 2007).

8. Zhang, S., Silver, J. M., Bi, T. & Del'Haye, P. Spectral extension and synchronization of microcombs in a single microresonator. *Nat. Commun.* **11**, 6384 (2020).

9. Moille, G. *et al.* Ultra-broadband soliton microcomb through synthetic dispersion. *arXiv: Optics* (2021).
